# Pharmacological therapy of neonatal analgosedation: current status, dilemmas, and perspectives

**DOI:** 10.3389/fped.2026.1757704

**Published:** 2026-03-25

**Authors:** Giacomo Cavallaro, Felipe Garrido, Nunzia Decembrino, Elena Emilia Bullejos Garcia, Genny Raffaeli

**Affiliations:** 1 Neonatal Intensive Care Unit, Fondazione IRCCS Ca’ Granda Ospedale Maggiore Policlinico, Milan, Italy; 2 Neonatal Intensive Care Unit, Clínica Universidad de Navarra, Madrid, Spain; 3 Neonatal Intensive Care Unit, Azienda Ospedaliera Universitaria Policlinico G. Rodolico San Marco, Catania, Italy; 4 Pediatric Anesthesia, Fondazione IRCCS Ca’ Granda Ospedale Maggiore Policlinico, Milan, Italy; 5 Department of Clinical Sciences and Community Health, University of Milan, Milan, Italy

**Keywords:** developmental pharmacology, intranasal sedation, neonatal pain management, neonatal sedation, opioid-sparing strategies, pharmacological analgesia, pharmacological sedation

## Abstract

Neonatal pain is now recognized as a critical issue, with evidence showing that even extremely preterm infants have nociceptive pathways and that untreated pain causes both short- and long-term problems. Despite greater understanding and better assessment tools, practices vary worldwide, with differences in guidelines, pain evaluation, and access to effective treatments. Opioids are vital for severe pain but are limited by side effects and uncertain long-term impacts. Alternatives such as acetaminophen, ketamine, and dexmedetomidine offer benefits, although evidence in neonates remains limited. Propofol provides rapid hypnosis but carries neurotoxicity risks and hemodynamic instability; midazolam is often used for anxiolysis, though concerns exist about its effectiveness, lack of analgesia, and adverse neurological outcomes. Benzodiazepines other than midazolam, such as lorazepam and diazepam, are used less frequently due to accumulation risks and benzyl alcohol toxicity. Intranasal formulations of fentanyl, midazolam, dexmedetomidine, and ketamine are emerging as quick, practical options for procedural sedation and analgesia, but more research is needed. Overall, neonatal pain management and sedation encounter gaps in evidence and practice, emphasizing the need for standardization, improved personalized approaches based on pharmacokinetic and pharmacodynamic maturation, multimodal strategies to minimize opioid and benzodiazepine exposure, and rigorous studies of new therapies to ensure safe, effective, and equitable care for newborns.

## Introduction

The recognition of neonatal pain and the need for appropriate sedation is now well established. Repeated painful exposure or inadequate sedation can alter cortical development, increase pain sensitivity, and contribute to long-term behavioral and neurodevelopmental effects (1). Despite advances in knowledge, clinical pain management and sedation practices remain inconsistent. Campbell-Yeo et al. report variability in the use of validated pain and sedation scales and clinical protocols, leading to disparities in access to adequate analgesia and appropriate sedation (2). Neonates continue to experience 7–17 painful procedures per day, often without proper analgesia or sedation, despite strong evidence for effective non-pharmacologic interventions such as skin-to-skin contact and oral sweet solutions (2, 3). Persistent gaps also exist in the implementation of personalized, multimodal treatment strategies that distinguish between analgesia for pain relief and sedation for anxiolysis or procedural tolerance (4).

The EUROPAIN study demonstrated substantial variability among European neonatal intensive care units (NICUs), with frequent use of opioids and sedatives without appropriate pain assessment or clear indication, a practice associated with hypotension, prolonged ventilation, and extended NICU stays (5). Critically, sedatives such as benzodiazepines are often administered without concurrent analgesia during painful procedures, reflecting confusion between sedation and pain management (5).

In Italy, two decades of national surveys document gradual but incomplete progress. Early assessments revealed limited adherence to procedural pain guidelines and inconsistent sedation practices, and more recent studies confirm persistent gaps in pain assessment, analgesic administration and appropriate sedative use during invasive procedures (4, 6–8). Globally, a 2025 survey of 98 countries identified three prescribing patterns for analgesics and sedatives driven by sociodemographic index (SDI) and geographical areas (9). Organizational factors, clinician training, and local protocols significantly influence practice (9). High-SDI settings follow evidence-based premedication practices more consistently, whereas low-SDI regions rely on less standardized methods, potentially overlooking the procedural benefits demonstrated by structured regimens that combine appropriate analgesia with targeted sedation (10, 11). Individualized, guideline-aligned strategies using validated pain scales are essential, especially during mechanical ventilation, where distinguishing between treating pain and facilitating ventilator tolerance is critical (12).

Necrotizing enterocolitis (NEC) highlights the complexity of neonatal pain and sedation. Although 94% of infants receive intravenous (IV) analgesia, many continue to show elevated pain scores and prolonged pain episodes, and sedatives are frequently co-administered without a clear rationale (13). Only 61% of units report having standardized protocols for NEC analgesia (14). The 2025 consensus recommended regular pain monitoring, heart rate variability assessment, and multimodal therapy for stage II or higher NEC, emphasizing careful use of sedatives only when clinically indicated and always in combination with adequate analgesia (15).

This review synthesizes current pharmacologic approaches to neonatal analgesia and sedation, examines the critical distinction between pain relief and anxiolysis, and evaluates emerging therapeutic options with attention to their neurodevelopmental safety profiles.

### Fundamental principles and the dilemma of the best drug

**Figure 1:**
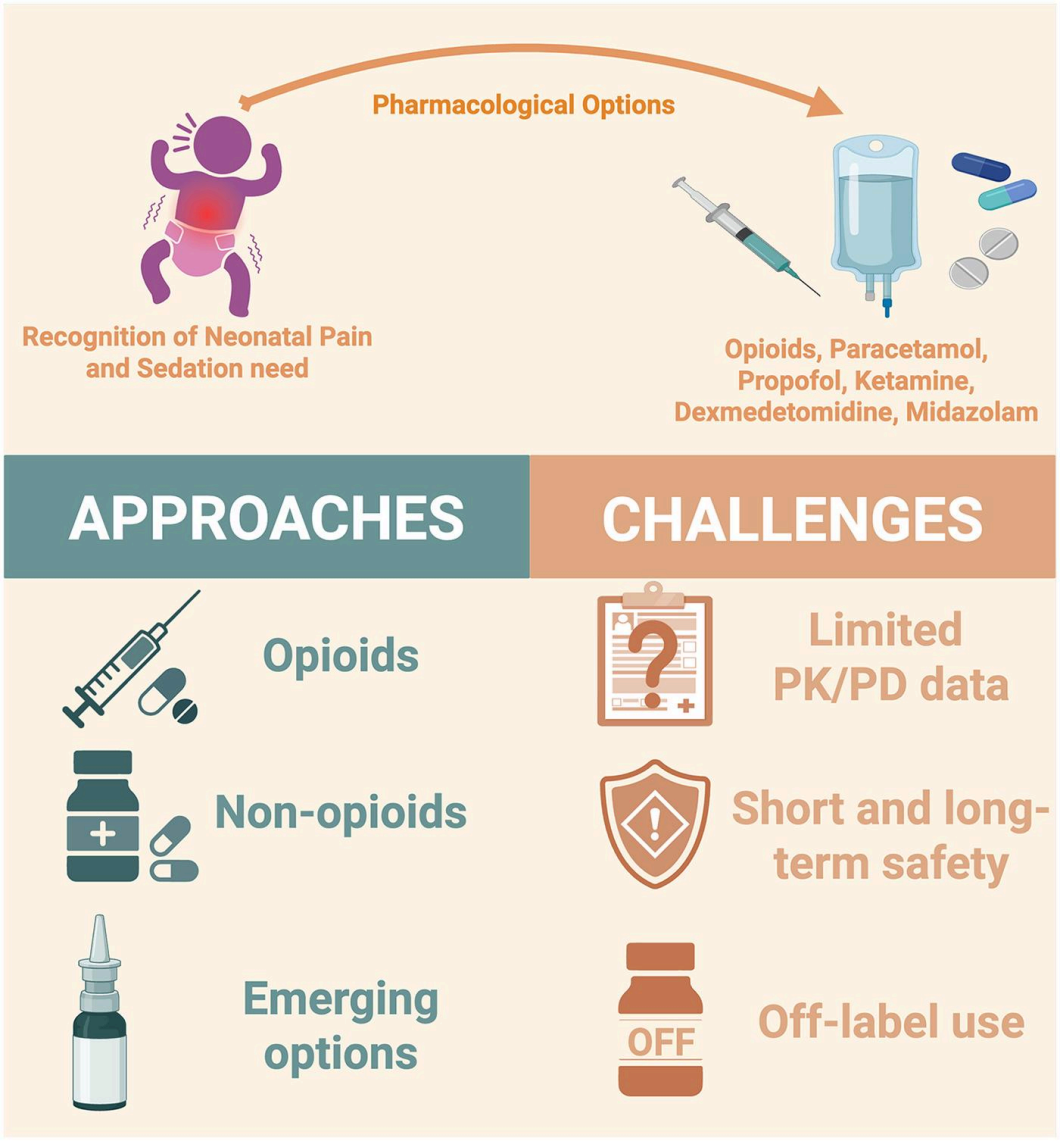
Current state in neonatal pain management: approaches and challenges. The figure outlines the clinical pathway from recognizing neonatal pain and sedation needs to available pharmacologic options, including opioids, non-opioids, and emerging therapies. It highlights current therapeutic approaches and key challenges in neonatal pharmacotherapy, including limited pharmacokinetic and pharmacodynamic data, uncertainties about short- and long-term safety, and widespread off-label use of many agents. Created at: https://BioRender.com.

Several core principles underpin safe and effective neonatal analgosedation practice and apply across all pharmacologic agents discussed in this review. Neonatal analgosedation drugs are widely used off-label, despite limited indications and specific dosing guidelines for neonates (16–26). Protocols and dosages are often adapted from pediatric or adult guidelines because of significant variability in pharmacokinetics (PK) and pharmacodynamics (PD). These differences require individualized dosing and close monitoring (17, 20). Choosing an appropriate analgesic or sedative treatment for a neonate is complex and often debated (27). Each medication class offers benefits but also carries significant risks, and treatment selection should be based on a personalized evaluation of the expected benefits and potential risks associated with each option (19, 28, 29). Currently, no pharmacologic agent can be considered “ideal” based on available evidence (26, 30). International literature suggests that medication choice, delivery method, and the use of drug combinations depend on the clinical context, neonatal maturity, and the availability of non-pharmacological options (Figure 1) (18, 20, 23, 24).

Treatment selection and titration must be guided by validated pain and sedation scales, applied systematically, and documented regularly (2, 31, 32). Objective assessment distinguishes pain from agitation, guides appropriate drug selection, and prevents both under- and over-treatment. In this context, combining non-pharmacologic interventions such as skin-to-skin contact, oral sweet solutions, and facilitated tucking with pharmacologic agents reduces drug exposure, improves efficacy, and minimizes adverse effects (33–35). Multimodal analgesia incorporating acetaminophen or regional techniques can substantially reduce opioid requirements (36–38).

A critical distinction must be made between analgesia and sedation: analgesics relieve pain, whereas sedatives reduce anxiety and promote tolerance of interventions but do not treat pain. Systematic reviews and randomized trials have not consistently shown benefits in clinically meaningful outcomes when opioids are routinely used for ventilated neonates without clear pain indicators (39–41). At the same time, sedatives should never be administered alone during painful procedures (5, 39, 41). Furthermore, PK and PD parameters vary widely with gestational age (GA), postmenstrual age (PMA), weight, and postnatal maturation of hepatic and renal function (17, 20, 42). Dosing considerations must account for these maturational changes, as well as non-maturational determinants, such as critical illness, organ or multi-organ failure, therapeutic hypothermia (TH), and extracorporeal circuit. Consequently, population PK (popPK) models support the development of personalized, maturation- or disease-aware treatment regimens (42–47).

Given the uncertainty about long-term neurodevelopmental outcomes and the potential for adverse effects, analgesics and sedatives should be used only when benefits clearly outweigh risks, at the lowest effective dose for the shortest necessary duration, with proactive weaning and withdrawal-prevention strategies (16, 18, 29, 48–50).

### Opioid drugs

#### Morphine

**Figure 2:**
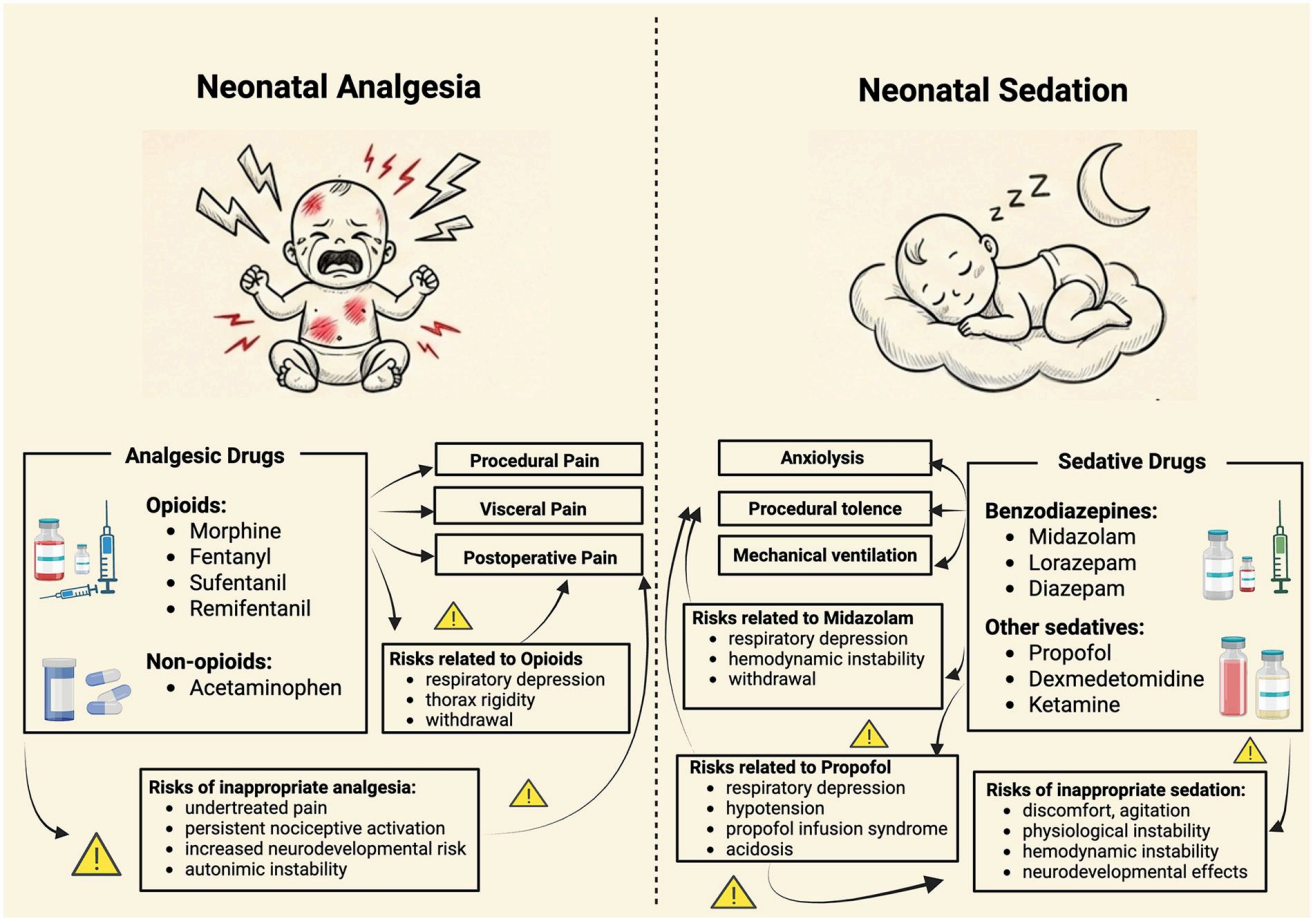
Neonatal analgesia versus neonatal sedation. The figure contrasts neonatal analgesia with neonatal sedation, highlighting their distinct clinical targets, pharmacologic agents, and risk profiles. Analgesia addresses procedural, visceral, and postoperative pain with opioid and non-opioid agents, emphasizing the risks of opioid exposure and inadequate pain control. Sedation targets anxiolysis, procedural tolerance, and facilitation of mechanical ventilation with sedative agents, each with specific drug-related risks and potential adverse consequences when misused. The schematic underscores that sedation does not provide analgesia and should never replace adequate pain management. Created at: https://BioRender.com.

Morphine, a hydrophilic opioid agonist with low lipid solubility, remains a crucial pain reliever in the NICU, although its dosing requires careful individualization (Table 1) (Figure 2) (42). Maturational shifts in enzyme capacity, renal clearance, body water, and protein binding alter exposure. The hepatic uridine diphosphate-glucuronosyltransferase 2B7 (UGT2B7) enzyme produces morphine-3-glucuronide (M3G), which mainly has excitatory effects rather than providing pain relief, and morphine-6-glucuronide (M6G), which is analgesic, with negligible renal excretion of unchanged morphine (42, 45). UGT2B7 activity in the newborn liver is estimated to be only 10%–20% of adult levels, and it does not reach adult levels until 2 months to 2.5 years of age. Doses appropriate at 28 weeks’ PMA often need adjustment later (42, 45).

**Table 1:** Practical, gestational Age-stratified dosing and monitoring framework for neonatal.

Opioids (Morphine, Fentanyl, Sufentanil, and Remifentanil).								
PMA	Indications	IV loading	Initial maintenance	Bolus	Titration & adjustments	Special situations	Monitoring & weaning	References
Morphine								
<28 wks	Analgesia for moderate–severe postoperative pain; not recommended for isolated procedural pain; not for primary sedation	50–75 μg/kg over 30–60 min	5–10 μg/kg/h	25–50 μg/kg q4–8 h	Reassess q2–4 h; adjust 10%–20% with validated scales and physiology	Renal impairment or hypothermia: reduce rate 25%–50% and extend intervals	Continuous cardio-respiratory monitoring; down-titrate for apnea/bradycardia, hypotension, hypercapnia; wean 10%–20% q12–24h when stable	(42, 45, 51–53)
28–31 wks	Analgesia for persistent or postoperative pain; limited role for procedural pain; sedation only as a secondary effect	75–100 μg/kg over 30–60 min	10–15 μg/kg/h	50–75 μg/kg q4–8 h	Anticipate rapid maturation with falling requirements	As above	As above	(42, 45, 51–53)
32–36 wks	Analgesia for postoperative pain or ongoing tissue injury; repeated procedural pain with cumulative burden, refractory to non-opioid measures; not a sedative agent	100 μg/kg over 30–60 min	10–20 μg/kg/h	75–100 μg/kg q4–6 h	Adjust 10%–25% steps per response	As above	As above	(42, 45, 51–53)
≥37 wks	Analgesia for postoperative and severe nociceptive pain; adjunct to multimodal strategies; ventilation *per se* is not an indication; use only if pain is present	100–150 μg/kg over 20–30 min	15–30 μg/kg/h	100 μg/kg q4–6 h	Higher starting clearance; avoid chasing plasma targets	Post-CPB: prioritize multimodal, opioid-sparing pathways	As above, favor earlier wean with effective non-opioids	(38, 42, 45, 52)
Fentanyl								
<28 wks	Analgesia is recommended for clearly painful conditions (e.g., postoperative pain, invasive procedures such as intubation or chest drain insertion). Not recommended for routine sedation or for improving ventilation tolerance in the absence of pain. Procedural pain is considered severe or repeated when it occurs within a multimodal strategy.	0.5–1 μg/kg over 10–20 min; consider an additional 0.5 μg/kg after 20–30 min if no effect; avoid rapid pushes.	0.5–1 μg/kg/h, start low	0.25–0.5 μg/kg q2–4 h	Reassess q2–4 h; change 10%–20% using validated scales and physiology; add non-opioids early.	Therapeutic hypothermia or organ dysfunction: reduce by 25%–50% and extend intervals; load cautiously.	Continuous cardiorespiratory monitoring; hold/down-titrate for apnea, bradycardia, hypotension, hypercapnia; wean 10%–20% q12–24h.	(44, 48, 70, 72, 80, 82, 308)
28–31 wks	Analgesia for postoperative pain and selected painful procedures. May reduce distress during invasive respiratory interventions when pain is present. Avoid use solely for sedation or ventilator synchrony.	0.5–1 μg/kg over 5–10 min.	0.5–1.5 μg/kg/h	0.5–0.75 μg/kg q2–4 h	Expect rapid maturational fall in need; plan step-downs over 24–48 h	nPICC in non-intubated infants: single 0.5–0.6 μg/kg with vigilant SpO_2_/CO_2_ monitoring	As above; predefine rescue ventilation plan if not intubated	(44, 70, 72, 82, 87–89)
32–36 wks	Analgesia for postoperative pain and for clearly painful procedures (e.g., intubation, ROP laser) within multimodal analgesia. Not indicated for routine procedural sedation or for ongoing ventilation tolerance without pain.	1 μg/kg over 5–10 min; for painful intubation, consider 2 μg/kg.	1–2 μg/kg/h.	0.5–1 μg/kg q2–3 h	Adjust by 10%–25%; without a load, infusion effect rises slowly over the first 12–24 h.	ROP laser: ∼1 μg/kg/h within multimodal plan; evidence mixed, high vigilance required.	Consider NIRS where available; small boluses typically do not perturb cTOI/output in stable infants.	(40, 70, 72, 74, 82, 84, 86, 90, 308)
≥37 wks	Analgesia for postoperative pain and painful procedures. It can be used as part of premedication for intubation. Routine use of mechanical ventilation without pain is not supported by outcome data.	1–2 μg/kg over 5–10 min; avoid rapid push.	1–3 μg/kg/h (use lower end if opioid-naïve).	1 μg/kg q2–3 h	Seek the lowest effective dose; schedule acetaminophen and other adjuncts for opioid-sparing.	Intubation premedication: improved conditions but possible BP drops; LISA: 1 μg/kg increased comfort; routine use in MV offers no outcome gain.	Continuous monitoring; structured taper 10%–20% q12–24 h; watch for urinary retention and withdrawal.	(16, 44, 48, 70, 82, 83, 87, 88, 309)
Sufentanil								
<28 wks	Analgesia only. Not indicated for routine procedural pain. It may be used for non-emergent intubation within structured premedication bundles or for selected severe postoperative pain. It is not indicated for sedation alone or for improving ventilation tolerance without pain.	Generally, avoid routine loading for continuous analgesia; reserve for severe pain or intubation.	0.02–0.06 μg/kg/h; centers reducing to ≤0.04 μg/kg/h.	Intubation: 0.1 μg/kg if ≤1,000 g.	Reassess q2–4 h; change 10%–20%	Organ dysfunction or hypothermia: reduce 25%–50% and extend reassessment intervals; ECMO: avoid reflex up-titration, re-score after circuit changes.	Continuous ECG/SpO_2_/capnography; hold or down-titrate for apnea, bradycardia, hypotension, hypercapnia; wean 10%–20% q12–24 h to limit withdrawal.	(50, 93, 101, 102, 104)
28–31 wks	Analgesia only. Indicated for premedication before intubation (non-emergent or semi-urgent) and for postoperative pain after major surgery. Avoid use solely for sedation or to prevent tolerance to ventilation.	No routine load for background infusion; consider tiny priming dose only if unstable procedural pain.	0.03–0.06 μg/kg/h start-low.	Intubation: 0.1 μg/kg if ≤1,000 g; 0.2 μg/kg if >1,000 g.	Adjust 10%–20%; add acetaminophen and non-opioid adjuncts early.	Transport or semi-urgent intubations feasible with sufentanil-based bundles but watch for rigidity/hypoxemia.	As above; predefine rescue ventilation if not intubated.	(50, 87, 93, 102, 104)
32–36 wks	Analgesia predominant. Indicated for preoperative intubation and for moderate–to–severe postoperative pain. May contribute to analgosedation only when pain is present.	For continuous sedation/analgesia, loading usually unnecessary; if needed, use small, slow bolus.	0.04–0.08 μg/kg/h	Intubation: 0.2 μg/kg common; avoid rapid push.	Adjust 10%–25%; without a load, infusion effect rises over 12–24 h.	ECMO expands Vd and may alter apparent CL; reassess clinically after circuit or support changes.	Consider NIRS where available; document pain/sedation targets each shift.	(42, 93, 101, 102, 104)
≥37 wks	Analgesia indicated. Procedural pain (planned intubatio) and moderate-to-severe postoperative pain. May be part of analgosedation strategies only if pain coexists. Sedation-only strategies without analgesia are inappropriate.	For planned continuous infusion in ventilated term neonates: ∼2 μg/kg load, then titrate, per PopPK proposal.	∼0.29 μg/kg/h model-based proposal; real-world ranges up to 0.72 μg/kg/h reported but start low and individualize.	Intubation: 0.2 μg/kg improves conditions within bundles; hypnotic-only strategies lack analgesia.	Seek the lowest effective dose; step-downs over 24–48 h as pain abates.	Conversions from other opioids are not adult-like; verify bedside effect when switching	Continuous monitoring; structured taper 10%–20% q12–24 h; watch urinary retention and withdrawal features.	(50, 69, 93, 102)
Remifentanil								
<28 wks	Procedural analgesia only for short, intense procedures requiring rapid onset/offset (e.g., endotracheal intubation, INSURE/LISA, emergent airway interventions). Not indicated for postoperative or ongoing pain. Sedation is limited to brief procedural facilitation only.	1–2 µg/kg over 45–60 s; avoid very rapid pushes	0.02–0.06 µg/kg/min (1.2–3.6 µg/kg/h), start at the low end	0.25–0.5 µg/kg every 2–3 min as needed	Reassess q1–3 min; change 10%–20% based on validated pain/comfort scales and physiology; prioritize non-opioid adjuncts	Hypothermia, severe illness, or co-sedatives: reduce by 25%–50% and lengthen reassessment windows; consider atropine if intubating	Continuous cardio-respiratory and capnography when feasible; watch for chest-wall rigidity; no taper required after brief use	(16, 70, 82, 115–117, 121, 310)
28–31 wks	Procedural analgesia for short invasive procedures with anticipated rapid recovery of spontaneous breathing (e.g., intubation, INSURE/LISA). It may be used as short-term facilitative sedation during brief mechanical ventilation. Not appropriate for postoperative analgesia.	1–2 µg/kg over 45–60 s	0.03–0.08 µg/kg/min (1.8–4.8 µg/kg/h)	0.25–0.5 µg/kg q2–3 min	As above; anticipate falling requirements with improving respiratory drive	For INSURE/LISA: pair with gentle positive-pressure support; avoid deep co-sedation	As above; if infusion >6–12 h, perform frequent wake-up checks to minimize exposure	(115–119, 121)
32–36 wks	Procedural analgesia for intubation, INSURE/LISA, nPICC placement, and other brief invasive procedures. Sedation is restricted to short-term use only and not indicated for postoperative or sustained analgesia.	1–2 µg/kg over 30–60 s; may repeat 0.5–1 µg/kg once if needed	0.04–0.10 µg/kg/min (2.4–6.0 µg/kg/h)	0.5–1 µg/kg q2–3 min if inadequate conditions	Adjust in 10%–20% steps; prefer short “test” increases and immediate down-titration after the noxious stimulus	nPICC placement or brief procedures: consider pure bolus strategy with minimal or no maintenance	Continuous monitoring; be prepared to assist ventilation; treat rigidity with brief neuromuscular blockade if required	(70, 115, 117, 118, 121, 310)
≥37 wks	Procedural analgesia for airway management, INSURE/LISA, and short painful procedures. It may be used as short-term sedation during brief ventilatory support. Not indicated for postoperative pain; transition promptly to longer-acting analgesics if ongoing pain is expected.	1–3 µg/kg over 30–60 s for intubation/LISA; slower if hemodynamically fragile	0.05–0.12 µg/kg/min (3.0–7.2 µg/kg/h), start low and titrate	0.5–1 µg/kg q1–2 min if needed	Rapid reassessment; down-titrate promptly once stimulus ends; transition early to non-opioid analgesia for visceral/ongoing pain	Airway procedures: increased risk of chest-wall rigidity with bolus dosing; ensure immediate availability of ventilatory support and neuromuscular blockade if required.	Continuous monitoring; due to ultra-short action, weaning is generally unnecessary; after prolonged use (>24–48 h) monitor for rebound discomfort rather than withdrawal	(82, 115, 118–120, 125, 311)

BP, blood pressure; CPB, cardiopulmonary bypass; CL, clearance; CO_2_, carbon dioxide; cTOI, cerebral tissue oxygenation index; ECG, electrocardiography; ECMO, extracorporeal membrane oxygenation; INSURE, intubation, surfactant administration, and extubation; IV, intravenous; LISA, less invasive surfactant administration; MV, mechanical ventilation; NIRS, near-infrared spectroscopy; nPICC, neonatal peripherally inserted central catheter; PMA, postmenstrual age; PopPK, population pharmacokinetics; ROP, retinopathy of prematurity; SpO_2_, peripheral oxygen saturation; Vd, volume of distribution; wks, weeks.

Classic neonatal cohorts demonstrate that parent morphine reaches steady state within 24–48 h, whereas M3G/M6G equilibrate more slowly; therefore, the overall effect may vary over multi-day treatments (51, 52). Clearance increases with GA and PMA, and the weak concentration–effect relationship supports titration to clinical efficacy, especially within the first 60 h (51, 52). Non-maturational determinants can reduce or alter clearance in critically ill term neonates (42).

**Figure 3:**
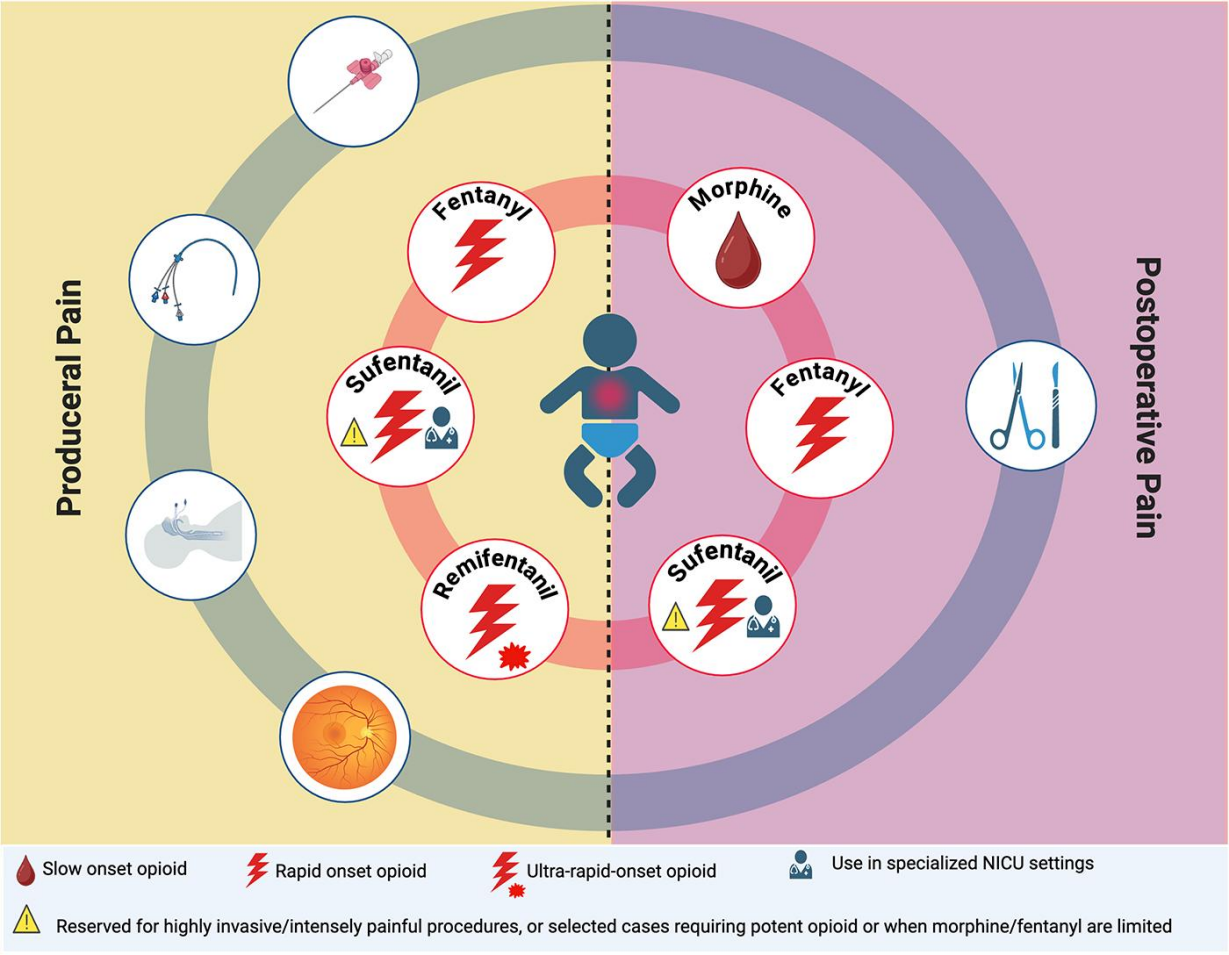
Opioids for procedural or postoperative pain. The figure illustrates the use of opioids in neonatal pain management, distinguishing procedural pain (left panel) from postoperative pain (right panel). Opioids are arranged by onset of action and clinical role: morphine, a slower-onset opioid used primarily for postoperative pain; fentanyl and sufentanil, rapid-onset opioids suitable for both procedural and postoperative pain; and remifentanil, an ultra–rapid-onset opioid mainly indicated for short, intensely painful procedures. Icons highlight situations that require specialized NICU settings and scenarios in which opioid use should be limited to highly invasive or carefully selected cases. The schematic underscores that opioid choice should be individualized based on the type and duration of pain, the anticipated recovery of spontaneous breathing, and the available monitoring and clinical expertise. Created in: https://BioRender.com.

Benefits are most evident for persistent pain, particularly in postoperative settings, where personalized dosing within multimodal approaches can reduce distress and enhance ventilator synchrony (Figure 3) (38, 42, 53). However, careful titration is necessary due to the risk of delayed clearance and accumulation (38, 51). Continuous infusion may be considered for persistent pain, though bolus dosing with frequent reassessments is often preferable in the most immature infants (18, 54).

In ventilated preterm infants, routine continuous morphine after a loading dose did not improve pain scores or neurologic outcomes compared with placebo, although a slight reduction in any grade of intraventricular hemorrhage (IVH) was observed (53). Similarly, the POPPI trial found that oral morphine failed to reduce pain scores or noxious-evoked brain activity and increased cardiorespiratory events (49). Therefore, routine morphine administration is not recommended in ventilated neonates without objective pain assessment or for short, repetitive procedural pain, such as heel lancing or endotracheal suctioning, in non-ventilated neonates (Table 2) (16, 18, 49, 52, 53, 55, 56). Non-pharmacological methods and short-acting analgesics are preferred to ensure efficacy and safety (18, 22, 34, 49, 56–60).

**Table 2:** Pros and cons of morphine, fentanyl, sufentanil, remifentanil, alfentanil, Acetaminophen, ketamine, propofol, dexmedetomidine, and midazolam in neonates.

Pros	Cons
Morphine	
Extensive NICU experience; effective for sustained, clinically meaningful pain when titrated to effect within multimodal pathways.	Weak concentration–effect coupling, large interindividual variability; metabolite lag (M3G/M6G) shifts net effect over days.
M6G contributes to analgesia; dosing can be individualized by GA/weight with loading then conservative maintenance.	Respiratory depression, hypotension, urinary retention, ileus; requires continuous monitoring and frequent re-assessment.
Can be used during therapeutic hypothermia with dose reduction and strict monitoring.	Ineffective and unsafe for brief procedures in non-ventilated preterms (increased cardio-respiratory instability); avoid routine use for short stimuli.
Useful as rescue within opioid-sparing post-operative strategies (e.g., paracetamol-based pathways).	Routine background infusions in ventilated preterms show no consistent analgesic benefit; prefer individualized, reassessed dosing.
Familiar hemodynamic profile; may improve ventilator synchrony when carefully titrated.	Reduced clearance with hypothermia/organ dysfunction/CPB; accumulation and potential withdrawal with prolonged infusions; unresolved long-term neurodevelopmental concerns mandate minimal effective dosing and follow-up.
Fentanyl	
Rapid onset, high potency, minimal histamine release.	Large interindividual variability; clearance driven by maturation and weight; need for loading; possible transporter/genetic contribution.
Low-dose boluses effective for nPICC in non-intubated infants with active monitoring.	ROP: analgesia often inadequate; higher instability vs. general anesthesia in several studies.
Effective analgesia; signal for reduced stress and neuron-specific enolase in some studies.	No consistent benefit on ventilation duration or mortality; avoid non-targeted routine use.
Small boluses generally do not alter cerebral tissue oxygenation index or ventricular output in stable preterms.	Intubation premedication can trigger transient blood-pressure drops; needs protocols and support.
Usable during therapeutic hypothermia with dose reduction.	Early continuous sedation may worsen diaphragmatic function in extremely preterm infants; favor minimal sedation.
Less early GI hypomotility than morphine; helpful when fast titration is required.	Urinary retention and withdrawal with prolonged infusions; chest-wall rigidity with rapid boluses.
Sufentanil	
Rapid central nervous system effect enables small-volume dosing and tight titration for procedures and postoperative visceral pain.	Risk of chest wall rigidity, desaturation, bradycardia without skilled airway support and vigilant monitoring.
Predictable lipophilicity; model-based dosing available in term neonates; accommodates short procedures and continuous infusions.	CYP3A ontogeny, inflammation, and ECMO alter exposure; conversions from other opioids are not what adult tables claim.
Low-dose strategies can control pain with fewer cumulative opioids after surgery.	Routine opioid infusion offers uncertain benefits on long-term outcomes or ventilation metrics; adverse effects accumulate with duration.
Remifentanil	
Ultra-short context-sensitive half-time enables minute-to-minute titration and fast recovery.	Chest wall rigidity with rapid/high bolus dosing; requires slow administration and readiness to ventilate.
Minimal accumulation; largely independent of renal/hepatic function.	Respiratory depression is concentration-dependent; continuous monitoring is mandatory.
Useful for brief procedures (intubation, LISA/INSURE, nPICC) with quick return of drive.	Not ideal as sole therapy for sustained visceral pain; needs multimodal adjuncts/transition plan.
Placental transfer clears quickly; induction-to-delivery time did not raise neonatal levels in cesarean-section cohort.	Evidence gaps on long-term outcomes and GA-stratified dose–response.
Alfentanil	
Rapid onset (1–2 min)	Nonlinear PK and prolonged elimination
Minimal accumulation	Hypotension, bradycardia, rigidity
Hemodynamic stability in older infants	High interindividual variability
Useful for brief procedures	Unsuitable for continuous sedation or NICU routine
Acetaminophen	
Opioid-sparing effect after surgery or sustained painful care; supports multimodal pathways prioritizing cardiorespiratory stability.	Limited efficacy for short, high-intensity procedural pain (e.g., ROP exam) when used alone; nonpharmacologic measures remain essential.
Predictable antipyresis; IV and enteral formulations allow route flexibility in fragile patients.	Narrow therapeutic window in extremely preterm infants due to immature conjugation and variable absorption; dosing must reflect PMA and weight.
Favorable hemodynamic profile compared with opioids; no histamine release.	Hepatic exposure increases with cumulative dosing; requires ALT/AST/bilirubin monitoring during prolonged courses.
NONMEM-informed dosing aligned to PMA can achieve analgesic targets while reducing morphine exposure	Evidence gaps for GA-stratified dose–response in the most immature; limited neonatal RCT data outside postoperative settings.
No renal adjustment needed; minimal interaction with renal replacement therapies.	Risk of inadvertent overdose from duplicate products (IV plus enteral) and from fixed pediatric schedules not adapted to neonatal maturation.
Ketamine	
Maintains airway tone and spontaneous ventilation	Potential neurotoxicity with prolonged exposure
Hemodynamic stability	Limited analgesia for deep visceral pain
Rapid onset, short duration	Variable PK in preterms
Bronchodilation, minimal respiratory suppression	Sparse long-term neurodevelopmental data
Propofol	
Rapid onset and short recovery for brief procedures.	Risk of significant hypotension and hemodynamic instability, especially in very preterm infants.
Predictable titration and a widely familiar agent in anesthesia settings.	No intrinsic analgesic effect — must be paired with analgesic adjuncts for painful procedures.
Short recovery profile potentially reduces time off ventilation or support.	Increased risk of Propofol Infusion Syndrome when used in high doses or prolonged infusion in neonates and children.
Less postoperative nausea/agitation vs benzodiazepines.	Respiratory depression, apnea, and need for airway support are increased risks in this population.
Well-studied PK/PD framework gives a basis for neonatal dosing research.	High inter-individual variability in clearance and volume of distribution due to immature metabolism (UGT, CYP pathways) — complicates dosing.
Dexmedetomidina	
Preserves spontaneous breathing and airway tone	Bradycardia and hypotension, usually reversible
Reduces opioid/benzodiazepine exposure	Inconsistent opioid-sparing effect post-surgery
Maintains stability during TH; neuroprotective potential	Alters cerebral activity (aEEG cycling, cTOI decrease)
Effective for procedural and regional anesthesia	Off-label use; requires continuous ECG and BP monitoring
Midazolam	
Rapid onset and effective sedation	High PK variability with risk of accumulation
Useful for ventilation tolerance and anxiety reduction	No intrinsic analgesia; must be combined with analgesics
Familiarity and extensive clinical experience	Potential withdrawal symptoms and urinary retention
Works within multimodal sedation strategies	RCTs in very preterm infants (24–32 weeks) showed a higher incidence of adverse neurological outcomes—including severe IVH and PVL—in midazolam-exposed neonates, though the specific risk for each lesion and for infants <32 weeks remains unquantified.
Hemodynamically stable in many scenarios	Interaction with co-medications (e.g., phenobarbital) alters clearance significantly

aEEC, amplitude-integrated electroencephalography; ALT, alanine aminotransferase; AST, aspartate aminotransferase; BP, blood pressure; CPB, cardiopulmonary bypass; cTOI, cerebral tissue oxygenation index; CYP, cytochrome P450; ECG, electrocardiogram; ECMO, extracorporeal membrane oxygenation; GA, gestational age; GI, gastrointestinal; INSURE, intubation, surfactant administration, and extubation; IV, intravenous; IVH, intraventricular hemorrhage; K, pharmacokinetics; LISA, less invasive surfactant administration; M3G, morphine-3-glucuronide; M6G, morphine-6-glucuronide; NICU, neonatal intensive care unit; NONMEM, nonlinear mixed-effects models; nPICC, neonatal peripherally inserted central catheter; PD, pharmacodynamics; PK, pharmacokinetics; PMA, postmenstrual age; PVL, periventricular leukomalacia; RCT, randomized control trial; ROP, retinopathy of prematurity; TH, therapeutic hypothermia; UGT, uridine diphosphate-glucuronosyltransferase.

Respiratory depression, hypotension, bradycardia, delayed gastrointestinal transit, urinary retention, and feeding difficulties are the main adverse effects of this opioid, particularly in premature infants due to their immature metabolism and clearance (18, 49). Long-term neurodevelopmental outcomes remain uncertain. Observational studies have reported associations between morphine exposure and poorer five-year cognition, although these findings are confounded by illness severity and the clinical indication for morphine use. Cochrane reviews conclude that the evidence regarding neurodevelopmental effects is very uncertain (16, 61–64).

Opioid stewardship data should incorporate opioid-sparing strategies and advise against using adult equianalgesic ratios for infants (19, 54, 65–67). After cardiopulmonary bypass, intermittent IV paracetamol reduced 48-hour morphine exposure by approximately 79% without worsening pain control, supporting opioid-sparing with rapid rescue (38). Long-term data are mixed; during TH for hypoxic ischemic encephalopathy (HIE), dexmedetomidine (DEX) and intermittent morphine produced broadly similar neurodevelopmental outcomes at 18–24 months (68). Moreover, a French NICU cohort reported markedly different infusion conversion ratios, and a systematic review found wide variability based on adult equianalgesic ratios, underscoring the need for infant-specific standards and mandatory re-titration after opioid switches (66, 69).

#### Fentanyl

Fentanyl is a valuable opioid in the NICU because of its rapid onset, high lipophilicity, and minimal histamine release (44, 70). However, given the wide variability in neonatal PK, conservative initial dosing and close monitoring are required (Table 2) (44, 70–72). Pooled popPK data indicate that both pre- and postnatal maturation support the use of a loading dose for rapid analgesia (44, 70–74). In neonates, fentanyl is mainly cleared by hepatic metabolism via cytochrome P450 (CYP) pathways (75, 76). CYP3A7 predominates early, shifting to CYP3A4 after birth (70, 72, 75–78). Maturational and non-maturational determinants modify clearance, so doses should be adjusted and reassessed regularly (72, 79, 80). Because fentanyl has a high hepatic extraction ratio, clearance depends more on hepatic blood flow than on enzyme capacity (16, 81). Renal excretion of unchanged fentanyl is negligible, and active metabolites do not significantly affect clinical outcomes (16, 71). These PK properties explain fentanyl's rapid onset, limited accumulation compared to morphine, and the need for GA-stratified dosing and frequent reassessment (72, 79, 82).

Fentanyl is primarily an analgesic opioid (Figure 2), although its potent effects are often accompanied by clinically significant sedation (Figure 3) (44, 83). In very preterm ventilated infants, continuous fentanyl during the first 72 h reduces behavioral pain and neuron-specific enolase without causing significant hemodynamic instability, suggesting possible neuroprotective effects when adequate analgesia is provided (81). However, routine opioid use does not improve ventilation duration, such as mortality, cardiac output, cerebral oxygenation, or neurodevelopmental outcomes, supporting selective, pain-guided use (Table 2) (16, 39, 41, 82, 83).

Fentanyl may be administered as a continuous infusion or intermittent boluses to provide sustained postoperative analgesia, especially in preterm infants, where hemodynamic stability is crucial (44, 84, 85). Rapid loading increases the risk of chest-wall rigidity; therefore, the initial bolus should be administered slowly (44, 70). In this context, fentanyl provides effective pain control with a more predictable time course than morphine when dosing is individualized and regularly reassessed (44, 72, 84, 85).

Fentanyl is effective for procedural pain in non-intubated infants. Its rapid onset makes it preferable to longer-acting opioids for acute procedures such as tracheal intubation and chest tube insertion (18, 82, 86). As premedication for tracheal intubation, fentanyl improves conditions but may cause a transient decrease in blood pressure, so protocolized support and rescue plans are essential (87, 88). Moreover, small fentanyl boluses are well tolerated during neonatal peripherally inserted central catheter (nPICC) placement with close monitoring (89). During laser therapy for retinopathy of prematurity (ROP), fentanyl infusion reduces behavioral pain compared to sucrose (84). However, larger and more recent studies have shown suboptimal effectiveness and increased cardiorespiratory instability compared to general anesthesia (Table 1) (85, 86, 90). Some authors discourage routine fentanyl use for ROP screening, recommending topical anesthesia, oral acetaminophen, and nonpharmacological measures instead (40, 91).

Fentanyl and morphine provide similar analgesia in neonates, but fentanyl is associated with fewer early gastrointestinal hypomotility events (77). Nevertheless, early continuous sedation may affect diaphragmatic ultrasound indices in extremely preterm infants, supporting the use of lower doses and shorter durations when possible (Table 2) (39). Long-term neurodevelopmental outcomes are inconsistent; some observational studies report associations with lower developmental quotients and impaired eye-hand coordination at 24 months, while systematic reviews find very low certainty evidence with variable effects, and substantial confounding by illness severity (64, 92).

#### Sufentanil

Sufentanil is a highly potent synthetic μ-opioid receptor agonist with high lipid solubility and rapid diffusion into the central nervous system (70). In full-term neonates, its PK profile is characterized by low clearance (∼0.651 L/h/kg) and a relatively large volume of distribution (4.7 L/kg), resulting in prolonged and variable exposure, particularly in preterm infants (42, 70, 93). Sufentanil undergoes extensive hepatic metabolism, primarily via CYP3A4-mediated oxidative N-dealkylation to inactive metabolites, with minimal renal excretion of the unchanged drug (70, 93, 94). Pharmacometric and physiologically based PK (PBPK) models support the notion that GA and body weight are weak but relevant covariates of sufentanil disposition, underscoring the need for individualized titration rather than fixed-dose schemes (93, 95–97). During extracorporeal support, the distribution and apparent clearance of drugs may increase due to sequestration, hemodilution, highly protein-bound and circuit binding (98–100). Therefore, score-guided adjustments are preferred over reflex up-titration (Table 1) (93, 101).

In mechanically ventilated neonates, sufentanil may contribute to analgesia regimens; however, its use should be driven by the presence of pain rather than solely to improve ventilator tolerance (Figures 2, 3) (16). Sufentanil, combined with midazolam and atropine, is generally effective for short procedures such as tracheal intubation; however, it can be complicated by rigidity, hypoxemia, and bradycardia, especially after multiple attempts (87, 102). The PRETTINEO trial reported that chest rigidity occurred in 13.8% of neonates receiving atropine, atracurium, and sufentanil, with four cases accompanied by bradycardia lasting more than 60 s (102). However, this regimen provided excellent sedation quality (92.6% vs. 51.7% with propofol) at the cost of a longer respiratory recovery time (102). In the NICU, opioid-containing regimens generally offer superior analgesia compared with hypnotic-only approaches. Moreover, follow-up data show no significant differences in neurodevelopmental outcomes between propofol-based and opioid-based premedication strategies, underscoring the need to balance analgesia, hemodynamics, and operator expertise (Table 2) (102, 103).

Continuous low-dose infusions effectively manage visceral and postoperative pain after patent ductus arteriosus (PDA) ligation, maintaining low pain scores without increasing adverse events and reducing cumulative exposure (104). Pharmacoepidemiology across 30 NICUs shows that administered doses of fentanyl and sufentanil exceed morphine when theoretical conversion ratios are applied (69).

Adverse effects of sufentanil in neonates are consistent with those of potent μ-opioid agonists and include respiratory depression, bradycardia, hypotension, chest wall rigidity, urinary retention, and gastrointestinal hypomotility (18, 48, 70, 102). Prolonged exposure and high cumulative doses are associated with withdrawal syndrome, and treatment duration is a major risk factor (50, 105). During intubation, rapid bolus administration increases the risk of rigidity and hypoxemia, particularly in very preterm infants (87, 102). Available data do not suggest a clear association between sufentanil exposure and increased rates of severe intraventricular hemorrhage, although vigilance remains warranted in vulnerable populations (106). Neurodevelopmental concerns warrant careful consideration. Although the evidence specifically for the therapeutic use of sufentanil in neonates shows no clear neurodevelopmental harm, emerging observational data on postnatal opioid exposure in infants undergoing cardiac surgery suggest associations with worse neurodevelopmental outcomes across cognitive, language, and motor domains at 2 years, though these associations are likely confounded by underlying cardiac disease severity and surgical complexity (102, 107). This underscores the importance of multimodal analgesia to minimize opioid exposure (33–35).

#### Remifentanil

Remifentanil is a potent μ-opioid receptor agonist with an ultra-short context-sensitive half-life, resulting from rapid hydrolysis by nonspecific plasma and tissue esterases (108–114). This pathway is independent of hepatic and renal function, leading to predictable clearance even in extremely preterm neonates and in the presence of multiorgan immaturity (108, 113–115). The primary metabolite, remifentanil acid (GR90291), is pharmacologically inactive and does not contribute to analgesic effects or accumulation (109, 115). PK studies reveal a small volume of distribution and high clearance, with maturational changes primarily driven by body weight rather than GA, resulting in relatively consistent offset times across neonatal subgroups (70, 108–114). Population-based target-controlled infusion models enable effect-site targeting using pediatric data; however, these models consistently emphasize that predicted concentrations must be clinically verified through validated pain and comfort assessments rather than used as stand-alone dosing tools (114, 116).

Compared with traditional opioids, remifentanil is suitable for short procedural analgesia rather than for ongoing analgesic needs, particularly during brief, intensely noxious procedures requiring rapid onset and offset with early recovery of spontaneous breathing, such as endotracheal intubation-surfactant-extubation (INSURE) or less-invasive surfactant administration (LISA) procedures, as well as brief invasive interventions (Tables 1, 2) (Figures 2, 3) (110–115, 117–119). In these settings, remifentanil provides adequate analgesia with minimal residual sedation, distinguishing it from longer-acting opioids such as morphine or fentanyl (110–114, 120). In contrast, remifentanil is not appropriate for postoperative pain management, as its ultra-short duration of action requires immediate transition to longer-acting analgesics to avoid rebound pain and physiological stress (82, 117, 120).

The most clinically relevant adverse effect is dose- and rate-dependent chest-wall rigidity, particularly after rapid bolus administration, which may compromise ventilation and necessitate immediate airway support or neuromuscular blockade (118, 120–122). Additional adverse effects include bradycardia, hypotension, and respiratory depression, particularly when remifentanil is combined with other sedatives or anesthetic agents (114, 120). Placental transfer occurs, but available data suggest that neonatal exposure following maternal administration is limited and rapidly cleared (123). Recent analytical studies using dried blood spot techniques confirm that neonatal plasma concentrations are typically low and transient, remaining below levels associated with sustained respiratory depression (124).

Long-term neurodevelopmental outcomes after neonatal remifentanil exposure are poorly understood. Evidence is currently limited to short-term procedural use, with no solid data available on cognitive, motor, or behavioral outcomes, underscoring the need for follow-up studies (22, 25, 26).

Practical recommendations emphasize slow titration, avoidance of large or rapid boluses, continuous cardiorespiratory monitoring in adequately equipped neonatal units, and proactive planning for alternative analgesic strategies when remifentanil is discontinued, particularly to prevent rebound discomfort after procedural use (119, 120, 125).

#### Alfentanil

Alfentanil is a short-acting opioid agonist metabolized by CYP3A4, with intermediate hepatic extraction and high affinity for *α*1-acid glycoprotein (AAG) (126, 127). Because neonates express only approximately 20% of mature CYP3A4 activity and have scant AAG, these developmental factors significantly reduce clearance, extending the elimination half-life to as much as 9 h. This reduced clearance, high protein binding, and nonlinear PK necessitate careful dosing adjustments in neonates to prevent accumulation and potential toxicity (70, 127–135). Although alfentanil provides adequate analgesia, its use entails significant benefit-risk considerations because of frequent side effects and procedural constraints identified in research. Specifically, the potential for thoracic rigidity, bradycardia, and transient desaturation is considerable, thereby limiting its use in the NICU (Table 2) (130, 132, 136, 137).

Additionally, early trials reported hypotension and desaturation after IV bolus, raising concerns about cerebral perfusion in premature infants (130, 132, 138). Currently, the Food and Drug Administration (FDA) does not recommend alfentanil for children under 12 years of age due to insufficient safety and efficacy data. Careful patient monitoring is essential, and the need for muscle relaxants should be considered whenever this drug is used in neonates (56, 136, 137). Long-term neurodevelopmental outcomes for alfentanil in neonates remain entirely uncharacterized. The lack of follow-up studies, combined with concerns regarding opioid-associated neurodevelopmental effects observed in animal models and observational human studies, necessitates cautious application and ongoing neurodevelopmental surveillance (18, 107, 139).

### Non-opioids drugs

#### Acetaminophen

Acetaminophen (paracetamol) acts centrally to provide analgesic and antipyretic effects by inhibiting the peroxidase site of prostaglandin H2 synthase in the central nervous system, rather than blocking peripheral cyclooxygenase (COX) (140, 141). Its analgesic effect is also mediated by active metabolites, such as N-arachidonoylphenolamine (AM404), which influence cannabinoid type 1 (CB1) receptors and transient receptor potential vanilloid-1 (TRPV1) channels in key pain control centers, including the periaqueductal gray and rostral ventromedial medulla (142, 143). These mechanisms account for its effectiveness in inflammatory and postoperative pain and explain the lack of sedative effects.

Neonatal PK changes rapidly after birth. Acetaminophen has a large volume of distribution at birth, and its clearance increases with body weight and PMA as hepatic conjugation matures (144, 145). In very preterm infants, sulfation is the primary pathway initially. Over the first few weeks, glucuronidation and oxidative pathways develop, thereby shortening the half-life and reducing drug exposure at the same dose (146, 147). Oxidative metabolism via CYP2E1 to N-acetyl-p-benzoquinone imine (NAPQI) is minimal in neonates and is efficiently detoxified by glutathione at therapeutic doses (148–152). Early studies of intravenous propacetamol support longer dosing intervals in extremely preterm infants (43, 153). Rectal administration in preterm neonates results in delayed absorption and a longer half-life (154). PK data from older pediatric groups should be used cautiously in neonates due to developmental differences in drug metabolism (155).

PopPK models consistently show that weight and maturity are the main predictors of acetaminophen exposure. This supports the use of a loading dose followed by maintenance dosing every 6–8 h to achieve target analgesic levels (144, 147, 152). Nonlinear mixed-effects modeling also supports maturity-adjusted dosing to optimize exposure and reduce variability (Table 3) (144, 147). Studies of prolonged use have shown predictable drug and metabolite levels, stable biochemical profiles, and no clinically significant hepatotoxicity in neonates treated for more than 72 h (156).

**Table 3:** Acetaminophen suggested dosing table.

Population	Route	Suggested regimen	References
≤30 weeks	Oral/rectal	∼25–30 mg/kg/day, q8–12 h	(145, 154, 158)
>30 to ≤34 weeks	Oral/rectal	∼45 mg/kg/day, q8 h	(145, 154, 158)
>34 to ≤36 weeks	Oral/rectal	∼50–60 mg/kg/day, q6–8 h	(145, 154, 158)
≥37 weeks	Oral/rectal	∼60 mg/kg/day, q6 h	(145, 158)
Preterm/term	Intravenous	∼20–40 mg/kg/day, q8–12 h in preterm; q6–8 h in term	(144, 146, 156)
NONMEM popPK models	Intravenous	Load ∼20 mg/kg, then ∼7.5–10 mg/kg q6–8 h; aim for consistent analgesic exposure via maturation-based adjustments	(144, 147, 156, 312)

IV, intravenous; NONMEM, NONlinear Mixed-Effects Models; popPK, population pharmacokinetics.

Acetaminophen is not a sedative and should not be used to promote ventilatory tolerance or behavioral suppression. Its clinical use in neonates is limited to analgesia, antipyresis, or PDA closure (Figure 2). As a first-line agent, it is indicated for neonatal fever and as an opioid-sparing adjunct in multimodal analgesia, with dosing adjusted for PMA and weight (157–159). For analgesia, it is essential to distinguish between procedural and postoperative pain. Analgesics are not routinely recommended for brief procedures such as heel lancing or ROP screening, where their effects are inconsistent and limited; nonpharmacological measures remain essential (160, 161). In contrast, acetaminophen reliably reduces opioid requirements and maintains comparable pain scores in postoperative neonates and young infants (Table 2) (36, 37).

Available neonatal follow-up data indicate a reassuring safety profile. Five-year follow-up studies from randomized trials show no increase in asthma, allergic disease, or other long-term morbidities after early neonatal exposure (162, 163). Specifically, neurodevelopmental assessments conducted at five years of age indicate no heightened risk of autism spectrum disorder, attention-deficit/hyperactivity disorder, or intellectual disability after neonatal acetaminophen exposure (162–164). Furthermore, concerns from epidemiological studies of prenatal exposure are primarily addressed by sibling-controlled analyses, which show no causal link between acetaminophen use during pregnancy and autism, attention-deficit/hyperactivity disorder (ADHD), or intellectual disability (165). Nevertheless, continued pharmacovigilance remains essential given the population's developmental vulnerability.

#### Ketamine

Ketamine is a noncompetitive antagonist of the N-methyl-D-aspartate (NMDA) receptor, providing dissociative anesthesia, pain relief, and sedation while maintaining airway reflexes and cardiovascular stability (7, 166). Its pharmacological profile makes it suitable for neonates, providing analgesia and amnesia without affecting breathing or blood pressure (167). However, a Cochrane review found only two low-quality randomized trials, making it difficult to establish firm guidelines (168).

PK data indicate a large volume of distribution, immature hepatic metabolism via CYP2B6 and CYP3A4, and a prolonged half-life in preterm infants (42, 46, 167, 169–172). Ketamine increases sympathetic tone and causes bronchodilation, unlike the respiratory depression caused by opioids or benzodiazepines (173, 174).

Ketamine has distinct indications for sedation and analgesia. It is used for short procedural sedation, such as intubation and ROP laser therapy (Figure 2) (90, 175–178). Studies show that it provides adequate analgesia and stable ventilation, and S(+)-ketamine, the better-tolerated enantiomer, reduces psychomimetic reactions and maintains airway tone (90, 176, 178–180). Its hemodynamic stability has been confirmed during cardiac procedures (169, 173, 178–181). Intramuscular ketamine provides effective anesthesia induction in neonates with difficult IV access, maintaining cardiorespiratory stability and achieving a rapid onset (182).

Although animal studies and organoid models demonstrate neurotoxicity with prolonged or repeated ketamine exposure, current human neonatal data do not show harm from single short exposures used for procedures such as intubation (167, 183–189). Animal studies show that ketamine's effects are context-dependent: it may be neurotoxic in the absence of nociception but neuroprotective in the presence of untreated pain (188). Elalouf et al. found no neurodevelopmental issues at 2 years in preterm infants who received ketamine during intubation, supporting Allegaert et al.'s advocacy for personalized dosing tailored to pharmacological maturity (167, 189). The clinical relevance of experimental neurotoxicity findings remains uncertain, as animal exposure patterns (prolonged, repeated, or high-dose) differ substantially from brief procedural use in human neonates (183–188). Additionally, some authors emphasize that ketamine should be administered for a short duration and at low doses, with continuous cardiorespiratory monitoring, to balance adequate analgesia with safety in neonates (190, 191).

Current evidence supports cautious use of ketamine in neonates until larger trials clarify optimal dosing, efficacy, and safety in perioperative and intensive care settings (Tables 2, 4) (168, 192).

**Table 4:** Practical neonatal ketamine, propofol, dexmedetomidine, and midazolam dosing framework.

Route	Dose	Indication	Notes	References
Ketamine				
IV bolus	0.5–1 mg/kg	Procedural pain/intubation	Combine with atropine to prevent bradycardia	(169, 175, 176)
IV infusion	0.5–2 mg/kg/h	Short sedation (<2 h)	Continuous hemodynamic monitoring	(169, 265)
IV bolus of S(+)-ketamine	1–2 mg/kg	ROP surgery, diagnostic procedures	Lower dose, fewer psychomimetic effects; limited evidence for continuous infusion in neonates	(179, 180)
IM	1.5–2.5 mg/kg *(max 3)*	Procedural or emergency analgesia (when IV not available)	Onset 3–5 min; duration 20–40 min; indicated when venous access is challenging; maintains cardio-respiratory stability; painful injection site	(182)
Propofol				
IV bolus	1.0–2.5 mg/kg over 20 s	Intubation/short procedures	Start low; monitor blood pressure ≥60 min	(200, 210, 211)
IV bolus	0.5–1 mg/kg over 20 s	With fentanyl or ketamine	Enhances stability and analgesia	(197, 199, 225)
IV infusion	1–4 mg/kg/h	Continuous sedation (off-label)	Avoid >1 h; monitor for acidosis	(212–215)
Dexmedetomidine				
IV bolus	0.3–1.0 µg/kg over 10 min	Imaging, intubation, short sedation	Avoid rapid push; monitor HR < 100 bpm	(17, 234, 247)
IV infusion	0.2–0.8 µg/kg/h	Continuous sedation in ventilated neonates	Adjust for GA, TH, and hemodynamics	(21, 227, 238)
IV infusion	0.2–0.5 µg/kg/h	under TH; HIE sedation	Improved comfort, opioid-sparing vs morphine	(241, 242, 245)
IV bolus+infusion	0.7–1.1 µg/kg + 1 µg/kg/h	Combined with caudal anesthesia, lower-limb or abdominal surgery	Stable sedation; limited hypotension	(247, 248)
Midazolam				
IV bolus	50–100 µg/kg	Adjunct for procedural sedation, imaging, or brief invasive procedures	Never analgesic alone; monitor for respiratory depression and hypotension; consider lower range in <32 weeks GA	(264)
IV infusion	30–60 µg/kg/h	Continuous sedation during mechanical ventilation	Adjust for GA, illness severity, CYP3A interactions; avoid accumulation with prolonged infusion; consider ∼30 µg/kg/h (0.03 mg/kg/h) in <32 weeks GA	(48)
IV infusion	20–40 µg/kg/h	Sedation during whole-body cooling for HIE	TH does not significantly change clearance; multimodal sedation recommended; monitor hemodynamics	(262)
IV Infusion	30–80 µg/kg/h	Postoperative sedation	Avoid prolonged exposure when feasible	(266)
IV Infusion	20–50 µg/kg/h	Adjunct infusion (with opioids or dexmedetomidine); Multimodal analgosedation to improve comfort and reduce opioid exposure	Synergistic effects; titrate with COMFORT/NPASS; monitor for cumulative sedation	(229)
Oral/Enteral (rare)	100–200 µg/kg	Pre-procedural anxiolysis when IV access is unavailable	Very high oral bioavailability in preterm infants; variable onset; use cautiously	(255)

CYP, cytochrome P 450; GA, gestational age; HIE, hypoxic ischemic encephalopathy; HR, heart rate; IM, intramuscular; IV, intravenous; NPASS, neonatal pain, agitation, and sedation scale; s, seconds; TH, therapeutic hypothermia.

#### Propofol

Propofol is a lipophilic intravenous hypnotic commonly used in NICUs for short procedures such as INSURE, LISA, and retinal laser therapy (Figure 2) (102, 193–197).

Neonatal PK is affected by the developmental maturation of hepatic enzymes such as UGT1A9, CYP2B6, and CYP2C9, with clearance increasing during the first postnatal weeks (193, 198–201). Hydroxylation is the predominant metabolic pathway in early neonatal life, whereas glucuronidation increases as UGT1A9 activity matures postnatally (119, 202). Modeling studies consistently show that standard allometric scaling tends to underestimate neonatal clearance, whereas using higher, age-appropriate exponents better reflects the faster elimination observed in early life (203–205). Additionally, clearance variability has been linked to bilirubin levels and postnatal age (198, 206). The GA- and postnatal age-dependent model emphasizes the importance of personalized propofol dosing strategies (207). Especially, hepatic perfusion and enzyme development play a larger role in propofol clearance than body weight alone (203–205).

Although propofol offers rapid hypnosis and predictable recovery, dose optimization is complex due to its immature metabolism and hemodynamic instability (119, 194, 208). Propofol induces hypnosis within 30–40 s and allows for recovery within 5–10 min, without intrinsic analgesic effects (209, 210). The NEOPROP trial defined an effective intubation dose; however, hypotension remains a common complication, affecting approximately one-third to nearly half of neonates within the first 24 h (102, 200, 210, 211). Moreover, continuous infusions exceeding 4 mg/kg/h or lasting more than 60 min increase the risk of Propofol Infusion Syndrome (PIS), which involves metabolic acidosis, rhabdomyolysis, and myocardial depression (212–215). Transient changes in amplitude-integrated electroencephalogram (aEEG) and near-infrared spectroscopy (NIRS), including brief cerebral desaturations, are typically reversible and not associated with long-term neurodevelopmental harm (201, 208, 216, 217). Animal studies and organoid models show that prolonged or repeated propofol exposure causes dose-dependent neuroapoptosis, learning difficulties, and disrupted microRNA (miRNA)-messenger RNA (mRNA) interactions (218–221). However, current human neonatal data do not show harm from single short procedural exposures, and short-term use appears safe up to two years of follow-up (201, 208, 216). The clinical relevance of experimental neurotoxicity findings remains uncertain, as animal and organoid exposure patterns differ substantially from brief procedural use in human neonates. However, prolonged or repeated exposures remain a concern (218–221). This safety concern has since led to a shift toward multimodal sedation strategies (222–224). Combining propofol with ketamine or fentanyl enhances sedation quality and hemodynamic stability while reducing the required doses (Tables 2, 4) (197, 215, 225, 226). Ongoing PBPK research aims to improve personalized dosing protocols (203–205, 207).

#### Dexmedetomidine

DEX has become a key part of pain management and sedation in NICUs, appreciated for its stable blood flow effects, possible brain protective properties, and minimal respiratory depression compared to opioids or benzodiazepines (23, 227, 228). By acting as a selective α2-adrenergic agonist at the locus coeruleus and dorsal horn, DEX provides calm, physiological sedation while decreasing pain signal transmission without affecting breathing (Figure 2) (82, 229). Its growing use shows a move toward opioid-sparing and neuroprotective approaches in neonatal care (125, 194, 230, 231). DEX use in NICUs has increased markedly since 2010, becoming one of the fastest-growing sedatives, despite limited neonatal labeling. A favorable safety profile has been observed in term neonates, particularly during cardiopulmonary bypass (232, 233).

DEX undergoes hepatic metabolism via UGT2B10 and CYP2A6, with significantly reduced clearance in preterm infants (234). TH further decreases metabolism, requiring individualized titration (235, 236). PK modeling shows wide variability among individuals based on GA and temperature (237). Curtis et al. confirmed hemodynamic stability, while Chrysostomou et al. validated dose-dependent safety (Table 4) (17, 234).

Clinically, DEX reduces opioid requirements and facilitates ventilator weaning, although postoperative outcomes remain inconsistent (238–242). Cochrane reviews support its safety during procedural and ventilatory sedation (21, 243). Emerging evidence suggests that DEX may exert neuroprotective effects during TH for HIE, thereby improving hemodynamic stability and reducing excitotoxic stress without impairing cerebral activity (241, 242, 244, 245). Additionally, DEX decreases stress and enhances comfort in preterm infants during short procedures (246, 247). Adverse drug reactions to DEX are primarily dose-dependent and mainly involve cardiovascular effects such as sinus bradycardia and mild hypotension, particularly during TH or following bolus administration (241–243, 248). More severe rhythm disturbances, including dose-related bradyarrhythmia, have been described in older infants after cardiac surgery (249). Neurophysiological investigations have revealed transient EEG slowing without concurrent desaturation or hypotension, suggesting that cerebral autoregulation is preserved despite reduced cortical activity (244). Prolonged infusions may occasionally lead to rebound hypertension or withdrawal irritability upon discontinuation, reflecting adaptive receptor changes after sustained exposure (250). Long-term neurodevelopmental outcomes indicate potential benefits; animal models demonstrate neuroprotection through mechanisms such as anti-inflammatory, anti-apoptotic, and autophagy-modulating pathways, all without impairing cognitive function. However, clinical validation in neonates with extended follow-up remains essential (21, 23, 227, 251).

Given these potential adverse responses, continuous ECG and invasive blood pressure monitoring are strongly recommended to promptly detect and manage hemodynamic fluctuations (230, 240, 252). Such vigilance is principally necessary when DEX is used alongside other sedatives or during complex perioperative or hypothermic care, where PD interactions may amplify cardiovascular sensitivity.

#### Midazolam

Midazolam remains widely used in NICUs despite uncertainties regarding its long-term safety (253). Midazolam functions as an anxiolytic but lacks analgesic effects; therefore, it should be combined with pain relievers during painful procedures or mechanical ventilation (Figure 2) (Table 4) (33, 254).

The metabolism of midazolam depends on CYP3A pathways, and significant developmental changes in these pathways affect clearance and exposure (82, 255). Extremely preterm infants have markedly reduced clearance, which increases with maturational progress, while phenobarbital further enhances clearance during TH (256, 257).

Modeling studies consistently show high interindividual variability and support personalized dosing across different GAs (258–260). Additionally, preterm infants have decreased intestinal CYP3A activity and high oral bioavailability, which limits the use of this medication via the enteral route (255). Further pharmacological research highlights how immature hepatic metabolism and concurrent therapies complicate drug handling, while case reports underscore the role of CYP3A polymorphisms in predisposing individuals to clinically significant drug accumulation (82, 261).

Clinical evidence remains limited, and Cochrane reviews show insufficient data on efficacy or safety (24, 253). Observational studies reveal substantial variability across clinical contexts, including postoperative care, mechanical ventilation, therapeutic hypothermia, and neonatal transport (5, 48, 87, 262). Excessive exposure may cause withdrawal, urinary retention, increased respiratory support, and prolonged hospitalization (48, 262, 263). Studies on premedication for intubation have demonstrated improved conditions; however, persistent desaturation and bradycardia persist (87, 264, 265). Midazolam remains widely used in postoperative NEC care, and comparative analyses indicate greater respiratory instability than with alternative sedatives (266, 267).

Trials in very preterm infants show higher rates of severe IVH and periventricular leukomalacia (PVL), despite a lack of lesion-specific or GA-stratified analysis (24, 253). This limited detail restricts understanding of whether the most immature neonates are more prone to neurological issues from midazolam use. Additionally, observational studies linking midazolam to adverse neurological outcomes often overlook confounding factors such as illness severity, ventilatory instability, and concurrent opioid use, making causal inference uncertain (5, 48, 106, 262). Cochrane reviews conclude that the evidence on neurodevelopmental effects is very doubtful (24, 253). Observational studies have reported associations between neonatal midazolam exposure and smaller hippocampal volumes, impaired working memory at age eight, and lower cognitive scores at 18 months. However, these associations may be confounded by illness severity and clinical indication for sedation (268–271). Preclinical models show dose-dependent synaptic changes (268). If its use is required, a reduced continuous infusion rate should be advised for infants with GA under 32 weeks, as their immature metabolic pathways slow drug clearance and place them at higher risk of accumulating excessive plasma concentrations during prolonged sedation or analgesic therapy (272). Using DEX as an opioid-sparing strategy may lower midazolam exposure in ventilated neonates, addressing limitations of midazolam-based regimens (229).

Midazolam enhances GABAergic inhibition during rapid synaptic development. Animal studies suggest that it may offer neuroprotective effects after hypoxic injury, such as decreasing inflammation and seizure burden; however, these effects remain unconfirmed in human neonates and are currently experimental (273).

These limitations highlight the need for detailed PK/PD studies and controlled trials to determine gestation-specific risk profiles and safer dosing strategies for extremely and very preterm infants (Table 2) (260, 272, 274, 275).

#### Benzodiazepines

Other benzodiazepines, such as diazepam and lorazepam, are used less frequently than midazolam in neonatology because of higher risks. Diazepam is typically used in minimal doses for seizure control. However, it is not recommended for neonates because they cannot metabolize it to inactive forms, leading to accumulation and prolonged central nervous system depression (128).

Lorazepam is often used for sedation, with dosing adjusted to minimize risk, though further evaluation is needed to determine the risk of severe neurotoxicity, particularly in neonates. Additionally, benzyl alcohol toxicity remains a significant concern, especially in preterm and low-birth-weight infants, as it may cause gasping syndrome, metabolic acidosis, and neurological complications (276).

Benzodiazepines lack analgesic properties and are often administered with opioids during painful procedures, increasing the risk compared with monotherapy. This combination increases the likelihood of respiratory depression and hypotension, potentially resulting in synergistic adverse effects (128, 276, 277).

When evaluating benzodiazepine outcomes in neonates, consider study limitations. A retrospective study of 63 neonates treated with lorazepam or midazolam found that 16% experienced adverse events, including seizures, hypotension, and respiratory depression. These events are likely linked to benzodiazepine use, but confounders such as illness severity and concomitant therapies may have influenced the results. This underscores the need to carefully assess study design when evaluating safety (277).

#### Nonsteroidal anti-inflammatory drugs

Non-steroidal anti-inflammatory drugs (NSAIDs) have shown promise in specific neonatal surgical settings (278, 279). However, their use in routine neonatal pain management remains uncertain due to limited and distinct clinical evidence (168).

NSAIDs inhibit COX enzymes, decrease prostaglandin production, and thus reduce peripheral and central sensitization to pain (280). PK data in neonates are available only for PDA closure; none are available for procedural pain indications (168). Ketorolac appears safe after neonatal cardiac surgery, showing stable renal function, no major bleeding, and reduced opioid needs, suggesting an opioid-sparing effect (279). Even in controlled settings, evidence for NSAID effectiveness in neonatal procedural pain remains insufficient, with no randomized trials addressing this major research gap (168).

### Emerging approaches and prospects

Intranasal formulations are becoming increasingly important for providing analgesia and sedation in neonates, especially when venous access is challenging, delayed, or unwanted. Fast systemic absorption through the highly vascularized nasal mucosa enables the avoidance of first-pass hepatic metabolism, resulting in relatively consistent PK, even in preterm infants (Table 5) (281).

**Table 5:** Neonatal Intranasal Drugs.

Drug	Dose	Indication	Notes	References
Fentanyl	1–2 µg/kg	Procedural pain; PICC insertion; palliative care	Onset 5–10 min; monitor for apnea in unstable infants; useful when IV access delayed	(282–285, 287–289)
Midazolam	100–200 µg/kg	MRI sedation; diagnostic procedures; non-emergent tracheal intubation	Onset 10–15 min; risk of desaturation in extremely preterm or unstable infants	(283, 290–292, 314)
Dexmedetomidine	2–3 µg/kg	MRI; ROP screening; non-invasive procedures	Onset 15–20 min; transient bradycardia possible; minimal respiratory depression	(82, 246, 290, 293–296)
Ketamine	2 mg/kg	Premedication for non-emergent intubation	Evidence from a single neonatal pilot study; faster onset vs. intranasal midazolam; PK/PD not established	(178)

IV, intravenous; MRI, magnetic resonance image; PICC, peripherally inserted central catheter; PD, pharmacodynamics; PK, pharmacokinetics; PMA, postmenstrual age; PVL, periventricular leukomalacia; RCT, randomized control trial; ROP, retinopathy of prematurity

**Figure 4:**
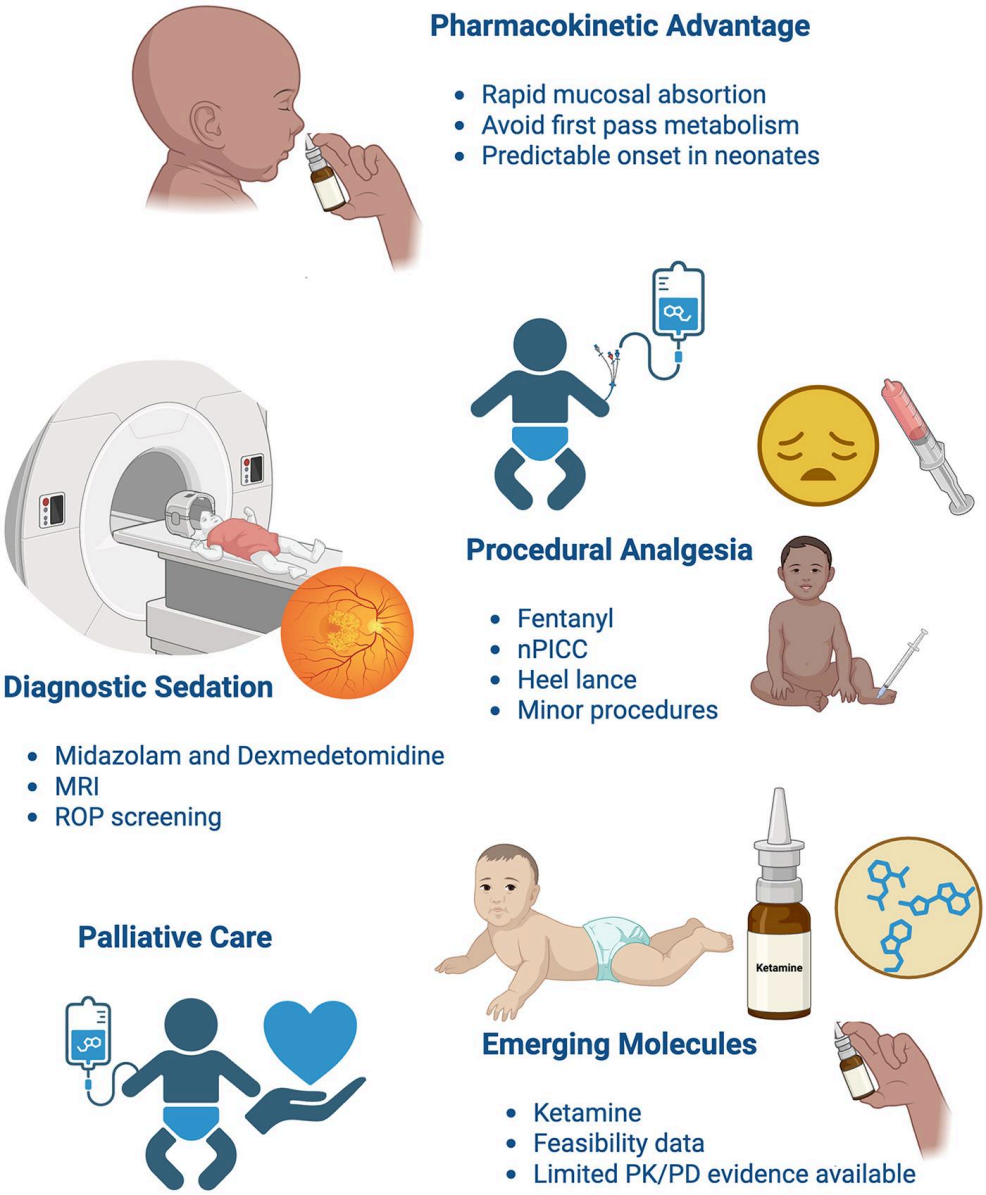
Intranasal formulations as emerging approaches. The figure illustrates how intranasal drug administration benefits neonates by facilitating rapid absorption, bypassing first-pass metabolism, and providing a predictable onset of action. It is used for procedural analgesia (e.g., fentanyl for heel lancing and nPICC), diagnostic sedation (e.g., midazolam and dexmedetomidine for MRI and ROP), and palliative care. Emerging drugs such as ketamine show promise, with some feasibility data, though pharmacokinetic and pharmacodynamic evidence in neonates remains limited. MRI, magnetic resonance imaging; PD, pharmacodynamic; nPICC, neonatal peripherally inserted central catheter; PK, pharmacokinetic; ROP, retinopathy of prematurity. Created at: https://BioRender.com.

Although neonatal PK/PD data are still limited, existing evidence shows that intranasal delivery can reach clinically significant drug levels with rapid onset and acceptable safety for fentanyl, midazolam, ketamine, and dexmedetomidine (Figure 4) (281).

Intranasal fentanyl has been studied in both procedural and palliative settings, showing rapid pain relief, good tolerability, and no significant respiratory problems, even in extremely preterm infants (282–289). Its lipophilicity allows for efficient mucosal absorption, making it a suitable alternative when intravenous opioids carry hemodynamic risks. Ongoing systematic studies aim to better understand early infant PK (286). Intranasal midazolam is often used for non-painful diagnostic procedures, such as MRI or tracheal intubation. Research in preterm and full-term neonates has demonstrated effective and predictable sedation, accompanied by stable heart and respiratory function (178, 283, 290, 291). It has also been shown to be feasible for retinal exams in preterm infants without causing physiological instability (292). Intranasal DEX provides reliable sedation without causing respiratory depression. Neonatal studies show that it is effective for MRI and ROP screening, decreases the need for rescue midazolam, and causes only transient, generally acceptable bradycardia, especially when lower doses are used (290, 293–296). Its sleep-like PD profile and minimal cardiovascular effects support its use in stable neonates, as well as in certain palliative or diagnostic situations (281). Evidence on the use of intranasal ketamine in neonates is limited; available reports suggest that it maintains hemodynamic stability and provides combined pain relief and sedation (178, 281). Most dosing and safety data are derived from studies in older children, and neonatal use should be primarily based on feasibility rather than extensive PK and PD research (25, 281, 297–299).

Overall, intranasal therapies are fast-acting, practical, and generally well-tolerated options for neonatal procedural and diagnostic care, with increasing evidence specific to neonates complementing broader pediatric experience (25, 297–306). Ongoing studies are needed to refine dosing, understand PK, and evaluate long-term safety.

## Conclusions

Recognition of neonatal pain has revolutionized NICU practices, emphasizing that even premature infants experience pain and that untreated stimuli can impede neurological development. However, care remains inconsistent due to the fragmented nature of guidelines and assessment tools. Effective management requires individualized approaches supported by organizational backing and continuous staff training (307).

Emerging agents such as ketamine and DEX show promise, with intranasal routes offering practical benefits; however, long-term evidence remains limited. Given the fragility of this population, neonatal analgosedation is a challenging yet vital pursuit for ethically sound, evidence-based care (167).
